# Addendum: Directing Min protein patterns with advective bulk flow

**DOI:** 10.1038/s41467-024-50256-6

**Published:** 2024-08-02

**Authors:** Sabrina Meindlhumer, Fridtjof Brauns, Jernej Rudi Finžgar, Jacob Kerssemakers, Cees Dekker, Erwin Frey

**Affiliations:** 1 Department of Bionanoscience, Kavli Institute of Nanoscience Delft, Delft University of Technology, Delft, the Netherlands; 2 Arnold Sommerfeld Center for Theoretical Physics and Center for NanoScience, Department of Physics, Ludwig-Maximilians-Universität München, Munich, Germany; 3 Max Planck School Matter to Life, Hofgartenstraße 8, 80539 Munich, Germany; 4 Present Address: Kavli Institute for Theoretical Physics, University of California Santa Barbara, Santa Barbara, CA 93106 USA

**Keywords:** Biological physics, Computational biophysics

Addendum to: *Nature Communications* 10.1038/s41467-023-35997-0, published online 27 Jan 2023

While the main message of our published paper^1^ – that the propagation direction of in vitro Min protein patterns can be controlled by a hydrodynamic flow of the bulk solution – remains unchanged, we discovered that the data of Fig. 2C in the paper cannot be used to support this point, due to an undersampling issue in the optical imaging.

When we performed the majority of experiments on how Min protein patterns react to the bulk flow, we set an imaging rate of 1 frame per 15 seconds in order to minimize the risk of photodamage from repeatedly imaging the same areas over hours of experimentation. This seemed a reasonable choice, as it captured the features of interest in preliminary experiments performed with MinE-wildtype, and it seemed adequate for the published (0.1–0.6 µm/s)^2^ and observed (up to 1.5 µm/s) values for the propagation speeds of Min protein patterns. For experiments with MinE-L3E/I24N^3^, we obtained velocities which seemed consistent with the results for MinE-wildtype, as shown in Fig. 2.

After the publication of the work, however, we repeated our experiments with MinE-L3E/I24N at higher sampling rates. These new data showed that the formerly chosen sampling rate under-sampled the speed of the patterns formed by this mutant. Fig. C1 shows examples of patterns acquired in new experiments with 1 µM MinD and 0.05 µM MinE-L3E/I24N. Images were now acquired at 1 frame per second. We find that the patterns travel over the length of one wavelength within roughly 20 seconds, meaning that the originally chosen imaging setting (1 frame per 15 seconds) was insufficient. Consequently, the original data for MinE-L3E/I24N in a flow, i.e. Fig. 2C and SI Figs. 27 and 31, were not suitable for reliably extracting the patterns’ propagation velocity.

**Fig. C1:**
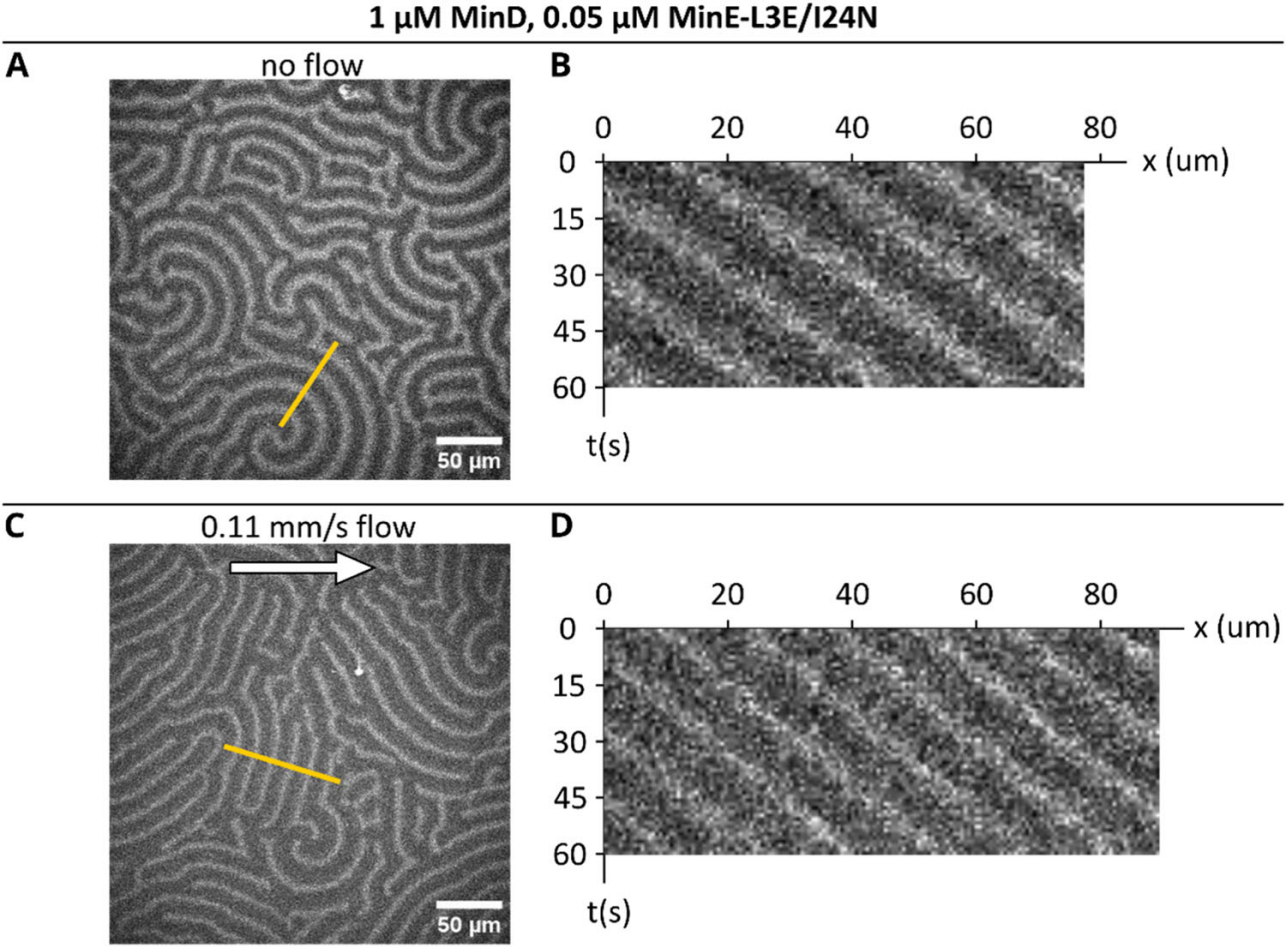
Min patterns for 1 µM MinD + 0.05 µM MinE-L3E/I24N. The Left shows a single frame; the right shows kymographs that were taken at the yellow line indicated on the left. **A** Snapshot of the pattern without flow. **B** Kymograph of designated line trace without flow. **C** Snapshot of pattern with 0.11 mm/s bulk flow. **D** Kymograph of designated line trace with 0.11 mm/s bulk flow. The flow was applied via the use of a syringe pump. The acquisition was started after a waiting time of at least 15 min.

By contrast, the originally chosen frame rate *was* sufficient to capture the propagation velocity of the patterns for the MinE-wildtype. Fig. C2 shows examples of patterns acquired in new experiments with 1 µM MinD and 5 µM MinE, now acquired at 1 frame per second. The data show the same result (a decreasing pattern velocity with flow) as in the published Fig. 4. To ensure this holds true for other MinE:MinD ratios as well, we performed new experiments for MinE-wildtype and performed a velocity analysis on them, as shown in Fig. C3. Based on this newly acquired data, we conclude that the sampling rate chosen for the original data was adequate to capture the features of interest for patterns formed by MinE-wildtype, and the data shown in the publication’s Fig. 4 (as well as associated SI figures) are not affected.

**Fig. C2:**
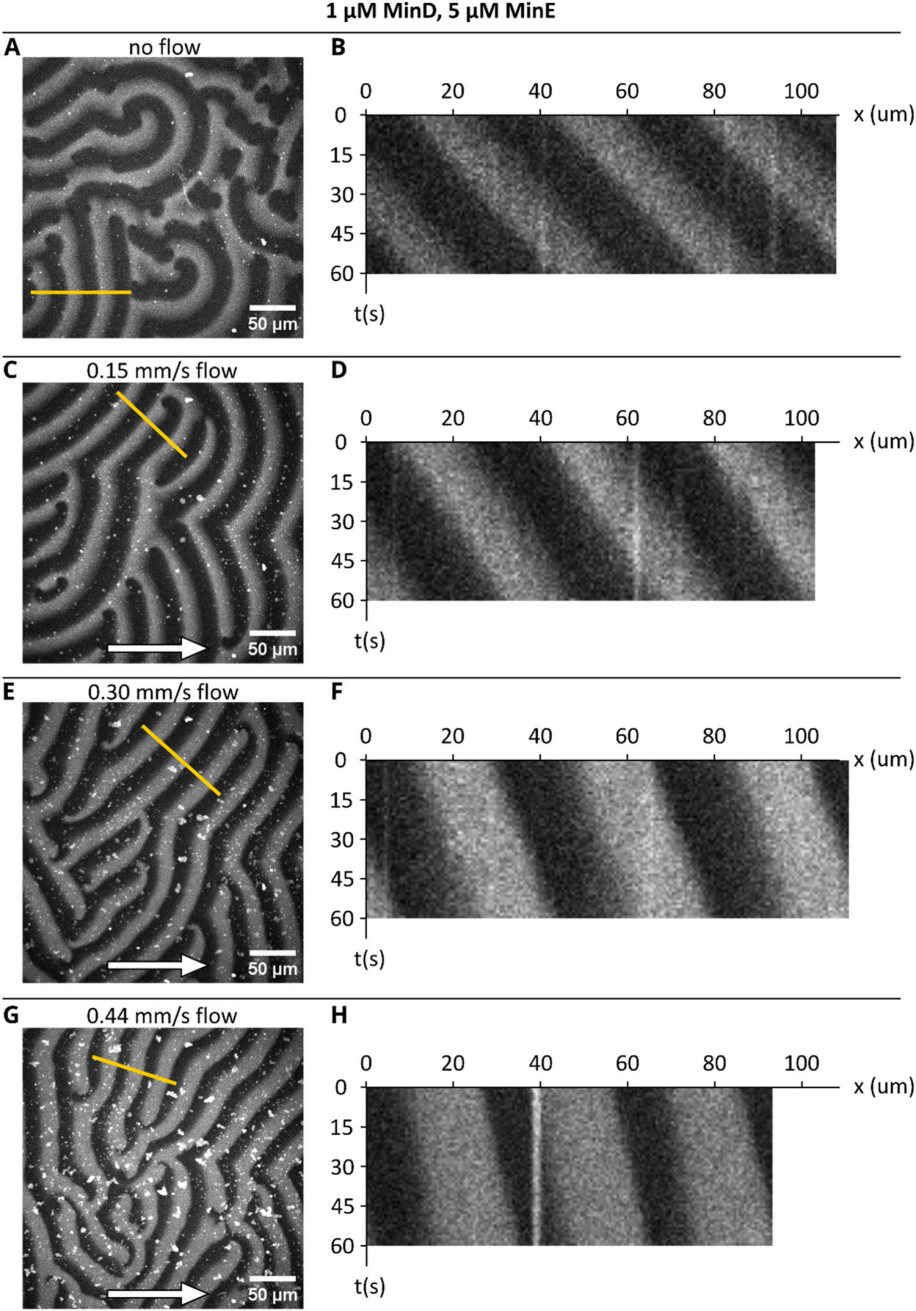
Min patterns for 1 µM MinD + 5 µM MinE. From top to bottom: Snapshots of pattern with **A** no flow, followed by increasing flow rates **C** 0.15 mm/s, **E** 0.30 mm/s, **G** 0.44 mm/s. Panels **B**, **D**, **F** and **H** show a kymograph at the line trace indicated in the corresponding image frame on the left. Flow was applied via a peristaltic pump through a closed-circle system. The acquisition was started after a waiting time of at least 15 min after changing the flow rate.

**Fig. C3:**
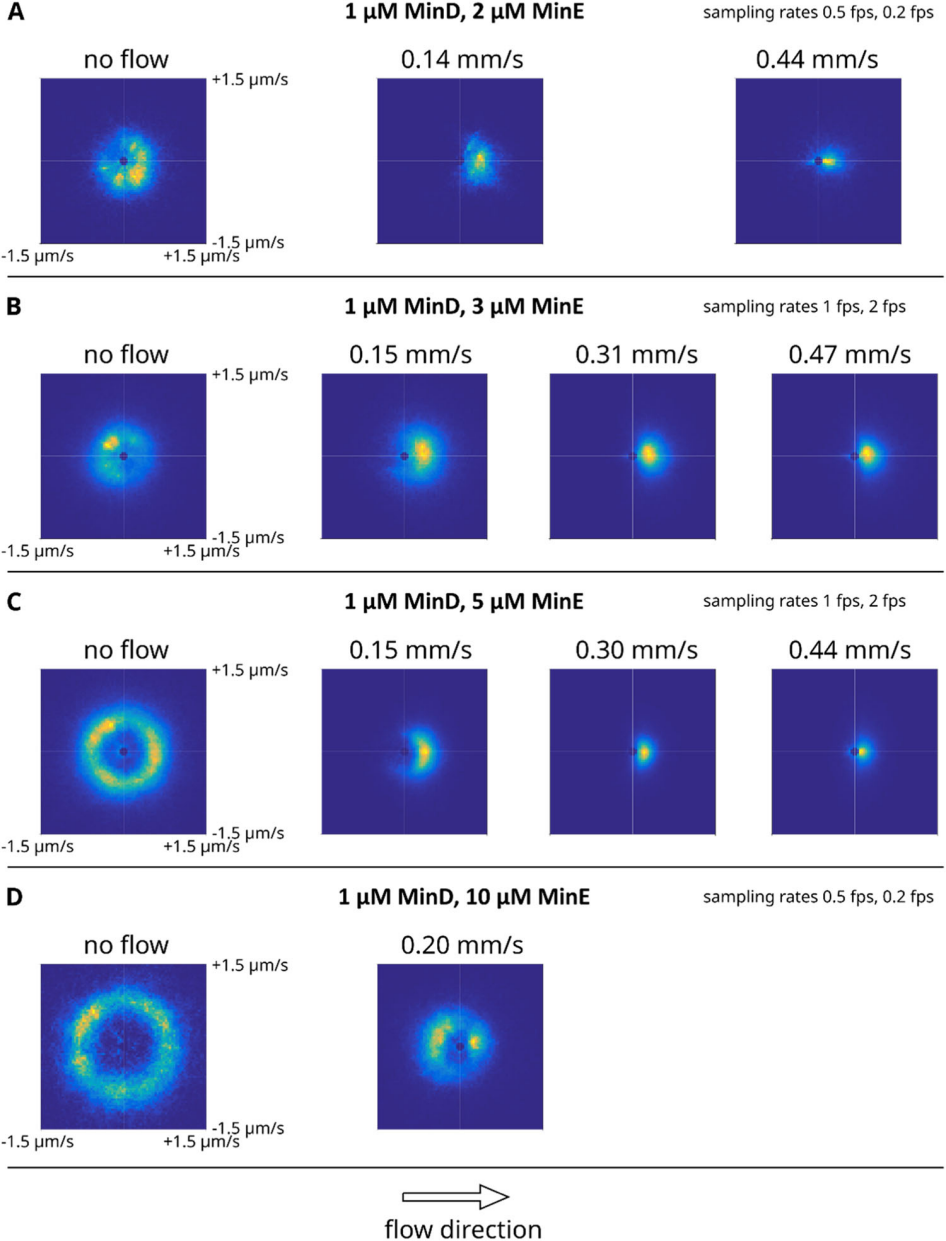
Repetition of experiments for MinE-wildtype with 1 µM MinD. **A** Data for 2 µM MinE. **B** Idem 3 µM MinE. **C** Idem 5 µM MinE. **D** Idem 10 µM MinE. Data shown are velocity analyses based on averaging over multiple individual field-of-views within the sample channel. Analysis was based on image data for MinD-Cy3.

Fig. C4 shows analysis results for additional experiments with MinE-L3E/I24N. Unfortunately, our report that MinE-L3E/I24N patterns only propagate downstream is not supported by these new data, as shown in Fig. C4 (though the initial directional trends already present in the absence of flow hampers an unambiguous interpretation of the automatized analysis). These new findings have implications for the modeling, as they indicate that the original data of Fig.2C cannot be used to support the skeleton model.

**Fig. C4:**
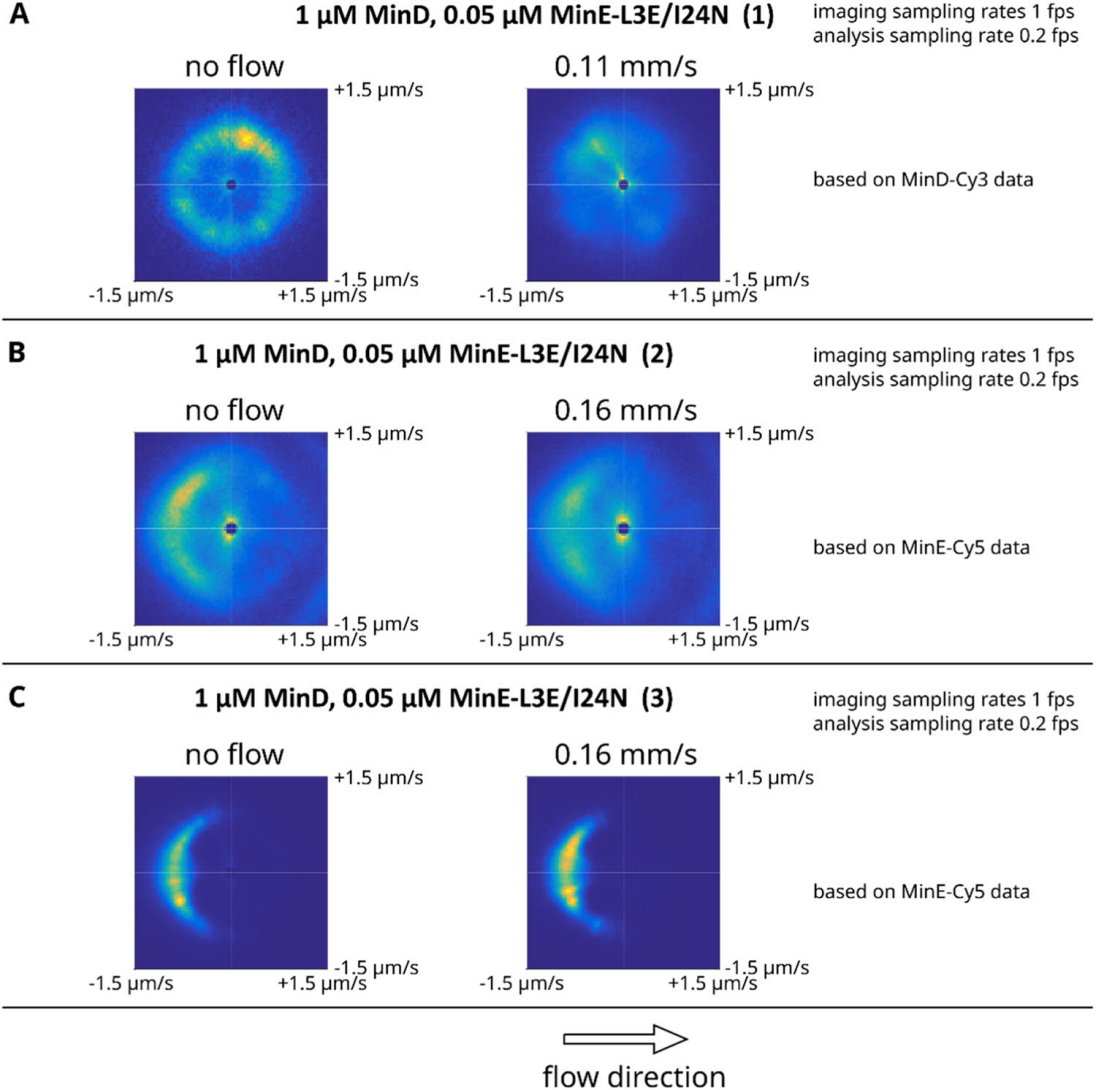
Repetition of individual experiments for MinE-L3E/I24N and associated velocity analysis, averaged over multiple individual field-of-views within the sample channel. **A**–**C** are three independent experiments.

In contrast to the statement made in the discussion of the manuscript, conformational switching still appears to be relevant even at low E:D ratios in the experiments. While there are possible candidates for molecular mechanisms not accounted for by the skeleton model that could be responsible for this discrepancy between theory and experiments, a conclusive answer has to await further experimental and theoretical studies.

The data associated with this correction can be found on: https://zenodo.org/records/11108536.
