# Supplementary material for: Directing Min protein patterns with advective bulk flow
Source: Nat Commun. 2023 Jan 27;14:450. doi: 10.1038/s41467-023-35997-0 (PMC9883515; doi:10.1038/s41467-023-35997-0)
Supplement: Supplementary file 3 — Description of Additional Supplementary Files [file 41467_2023_35997_MOESM3_ESM.docx]

**Description of Additional Supplementary Files**

**File Name: Supplementary Movie 1**

**Description:** Simulation result showing upstream propagation, duration 50 s, nE =1000 /μm2, vf = 50 μm/s.

**File Name: Supplementary Movie 2**

**Description:** Simulation result showing downstream propagation, duration 50 s, nE = 150 /μm2, vf = 50 μm/s.

**File Name: Supplementary Movie 3**

**Description:** Experimental results for E:D=10, MinE wildtype, without flow, 0.11 mm/s, 0.23 mm/s and 0.23 mm/s reversed.

**File Name: Supplementary Movie 4**

**Description:** Experimental results for E:D=2 (corrected 1.3), MinE wildtype, without flow, 0.21 mm/s, 0.42 mm/s and 0.63 mm/s.

**File Name: Supplementary Movie 5**

**Description:** Experimental results for E:D=0.05, MinE L3E/I24N, without flow and 0.15 mm/s.

**File Name: Supplementary Movie 6**

**Description:** Simulation result showing upstream to downstream transition, duration 600 s, nE = 191.4 /μm2. Flow velocity vf decreases linearly from vf = 100 μm/s at t = 0 s, to vf = 50 μm/s at t = 100 s, remaining at that value until the end of the simulation.
